# Protocol for development of the guideline for reporting evidence based practice educational interventions and teaching (GREET) statement

**DOI:** 10.1186/1472-6920-13-9

**Published:** 2013-01-25

**Authors:** Anna C Phillips, Lucy K Lewis, Maureen P McEvoy, James Galipeau, Paul Glasziou, Marilyn Hammick, David Moher, Julie Tilson, Marie T Williams

**Affiliations:** 1 School of Health Sciences, University of South Australia, GPO box 2471, Adelaide 5001, Australia; 2 Health and Use of Time Group (HUT), Sansom Institute for Health Research, University of South Australia, GPO Box 2471, Adelaide 5001, Australia; 3 International Centre for Allied Health Evidence (iCAHE), School of Health Sciences, University of South Australia, GPO Box 2471, Adelaide 5001, Australia; 4 Ottawa Hospital Research Institute, Centre for Practice-Changing Research, The Ottawa Hospital, 501 Smyth Rd, Ottawa, Ontario K1H 8L6, Canada; 5 Centre for Research in Evidence-Based Practice (CREBP), Bond University, University Drive, Robina, Queensland 4226, Australia; 6 Bournemouth University, Royal London House, Christchurch Road, Bournemouth, Dorset, UK; 7 Ottawa Hospital Research Institute, Ottawa, Canada; Department of Epidemiology and Community Medicine, Faculty of Medicine, University of Ottawa, Ottawa, Canada; 8 University of Southern California, Division of Biokinesiology and Physical Therapy, 1540 Alcazar St, CHP155, Los Angeles 90089, USA; 9 Nutritional Physiology Research Centre (NPRC), School of Health Sciences and School of Population Health, University of South Australia, GPO Box 2471, Adelaide 5001, Australia

**Keywords:** Evidence based practice, Education, Reporting guideline

## Abstract

**Background:**

There are an increasing number of studies reporting the efficacy of educational strategies to facilitate the development of knowledge and skills underpinning evidence based practice (EBP). To date there is no standardised guideline for describing the teaching, evaluation, context or content of EBP educational strategies. The heterogeneity in the reporting of EBP educational interventions makes comparisons between studies difficult. The aim of this program of research is to develop the **G**uideline for **R**eporting **E**BP **E**ducational interventions and **T**eaching (GREET) statement and an accompanying explanation and elaboration (E&E) paper.

**Methods/design:**

Three stages are planned for the development process. Stage one will comprise a systematic review to identify features commonly reported in descriptions of EBP educational interventions. In stage two, corresponding authors of articles included in the systematic review and the editors of the journals in which these studies were published will be invited to participate in a Delphi process to reach consensus on items to be considered when reporting EBP educational interventions. The final stage of the project will include the development and pilot testing of the GREET statement and E&E paper.

**Outcome:**

The final outcome will be the creation of a **G**uideline for **R**eporting **E**BP **E**ducational interventions and **T**eaching (GREET) statement and E&E paper.

**Discussion:**

The reporting of health research including EBP educational research interventions, have been criticised for a lack of transparency and completeness. The development of the GREET statement will enable the standardised reporting of EBP educational research. This will provide a guide for researchers, reviewers and publishers for reporting EBP educational interventions.

## Background

Evidence Based Practice (EBP) is a universal philosophy for decision making in health care which considers the patient’s perspective, therapist expertise and best available research evidence [1]. It has been proposed that EBP consists of a five step process with the first four steps involving asking a clinical question, acquiring and appraising the evidence, and applying the evidence into clinical practice. The fifth step encourages individuals to reflect upon the process undertaken in the first four steps [1,2].

 The inclusion of EBP education in entry-level training is an accreditation requirement for many health professional disciplines [3,4]. The Sicily consensus statement on EBP [2] recommended that EBP curricula should be grounded in the five step model, the efficacy and effectiveness of teaching each step should be researched (underpinned by systematic reviews) and that courses claiming to teach EBP should evaluate each step using validated assessment tools. In response to the increasing number of instruments available to assess various aspects of EBP, a second Sicily statement has recently been published [4]. This statement presents the ‘classification rubric for EBP assessment tools in education’ (CREATE) framework to provide guidance and recommendations for developers of educational instruments. One of the intentions of the CREATE framework is to allow “comparisons across studies by using a common set of outcome tools” [4]. The framework also provides information concerning classification of instruments based upon the five EBP steps, assessment category and intent, intended audience and pedagogy. The publication of a second Sicily statement reflects the rapid evolution of EBP and the need for standardised reporting processes.

A number of systematic reviews of EBP educational interventions have commented on the lack of detail provided in the reporting of EBP educational interventions [5-9]. The value of standardised guidelines for reporting research processes is well accepted, with more than 200 reporting guidelines currently available for health research [10]. These include randomised controlled trials (RCT) (n = 14) [e.g. the CONsolidated Standards of Reporting Trials (CONSORT)] [11], systematic reviews with or without meta analyses (n = 3) [e.g. Preferred Reporting Items for Systematic Reviews and Meta-Analyses (PRISMA)] [12], observational studies (n = 30) [e.g. Strengthening the Reporting of Observational Studies in Epidemiology (STROBE)] [13] and non-randomized trials (n = 7) [e.g. Transparent Reporting of Evaluations with Nonrandomized Designs (TREND)] [14]. To the best of our knowledge no reporting guideline exists which presents the key items to be considered when describing educational interventions for teaching foundation knowledge and skills of EBP.

 Recently, recommendations for the phases necessary to develop a reporting guideline in health research have been published [15]. The development process planned for this research program is based on these recommendations.

 The proposed reporting guideline for EBP educational interventions is planned to be used in conjunction with, rather than to replace reporting guidelines appropriate for the specific research design (CONSORT, STROBE, TREND etc.). This reporting guideline has the potential to benefit a number of education, health and research stakeholders. The guideline will provide detailed information to assist authors developing manuscripts of EBP educational interventions, peer reviewers and journal editors when reviewing such manuscripts. The guideline could also result in increased consistency in reporting of educational interventions, which could allow consumers of research to directly compare the educational interventions between studies and assist the undertaking and reporting of systematic reviews. Finally, the proposed guideline could be valuable for curriculum developers to consider when designing EBP courses.

In this proposal, an EBP educational intervention incorporates the teaching of knowledge and skills recommended in the five steps of EBP (Sicily statement 1) [2]. It does not include educational interventions designed for advanced or practitioner knowledge of the best evidence underpinning specific health care interventions or management strategies. The proposed study does not intend to develop a guideline or recommendations for research approaches (study designs), for assessing the efficacy of EBP educational interventions, educational strategies for teaching, learning or assessing EBP, or the specific syllabi / curricula for teaching EBP. The aim of this research project is to develop a **G**uideline for **R**eporting of **E**vidence based practice **E**ducational interventions and **T**eaching (GREET) statement and E&E paper.

## An explanation of the terms used in this proposal

### Doctoral Panel

This four member panel consists of the Principal Investigator (AP) currently undertaking this program of research as part of a Doctor of Philosophy in Health Science (PhD), University of South Australia and the supervisory team consisting of the principal supervisor (MTW) and associate supervisors (MPM, LKL).

### Expert Panel

Experts with prior knowledge and experience in EBP educational theory, authors from the two pre-existing Sicily statements [2,4], authors responsible for the development of reporting guidelines and the dissemination of scientific information.

### Research Team

The combined Doctoral and Expert Panels.

### Delphi participants

Participants in the Delphi consensus survey.

### EBP educational intervention

An educational strategy used to facilitate knowledge and skills in the five steps of EBP; asking a clinical question, acquiring and appraising the evidence, applying the evidence into clinical practice and reflecting upon the process undertaken in the first four steps (Sicily statement 1) [2].

## Methods

The research program will be undertaken in three stages:

1. Systematic review of EBP educational interventions.

2. Delphi survey to gain a consensus opinion on items to be considered within a standardised reporting guideline for EBP educational interventions.

3. Development and pilot testing of the draft GREET statement and accompanying E&E paper.

An Expert Panel of five members has been convened to review and guide each of the three stages of the program. Members of this panel currently include Dr Paul Glasziou (co-author of Sicily 1), Dr Julie Tilson (author of Sicily 2), Dr David Moher (author of reporting guidelines) and Dr James Galipeau (founder of a centre to study scientific writing and publications), and Dr Marilyn Hammick [Consultant to Best Evidence Medical Education (BEME)].

The role of the Expert Panel will be to:

1. Review and finalise the proposed research protocol.

2. Monitor the systematic review, identify any further relevant articles for inclusion, review and contribute to the manuscript reporting the results of the systematic review (stage 1).

3. Monitor and review the results of each round of the Delphi and contribute to the manuscript for disseminating the results of the Delphi survey (stage 2).

4. Guide and facilitate the process used to develop and pilot test the GREET statement and E&E paper including the method of communication (email, internet site), likely number of meetings, method of feedback and method of resolving disagreements for drafting of the GREET statement (stage 3).

5. Review, finalise and contribute to the dissemination of the GREET statement and E&E paper.

## Stage 1 - systematic review

This systematic review aims to describe how EBP educational interventions have been reported in controlled studies investigating the efficacy of EBP training/education.

The proposed systematic review question is:

‘What specific information has been reported when describing educational processes used in EBP educational interventions?’

### Databases and search terms

A preliminary systematic review protocol was drafted based on previous systematic reviews in the area of EBP and education [6,8,16-19], the evidence-based practice guidelines for the peer review of electronic search strategies [20] and the guidelines provided in the PRISMA statement [12]. Modifications were made following peer review of the draft search strategy protocol by members of the international Centre for Allied Health Evidence (iCAHE) and two academic librarians [20]. The proposed databases and search terms (including all terms and medical subject headings relating to EBP, education and health professional disciplines) are presented in Table 1. The search aims to retrieve original studies of EBP educational interventions, limited to participants aged over 18 years without limitations placed on language of publication or publication date. At this stage no limits were placed upon study design.

**Table 1:** **Draft search strategy plan**[6-9,16-19,21]

**Databases (search from inception)**	**Search terms**
MEDLINE	(1) Health or medic* or nurs* or “allied health” or physiotherap* or “physical therap*” or “occupational therap*” or “speech therap*” or “speech patholog*” or diet* or nutrit* or “social work*” or psycholog* or podiatr* or “ambulance paramedic” or “ambulance officer” or “music therap*” or “art therap*” or osteopath*or chiropract* or dentist* or dental* or optometr* or “medical radiation*” or “medical radiation science*” or radiograph*or pharmac* or “exercise physiolog*” AND
Academic Search Premier	
ERIC	
CINAHL	
Scopus	(2) Instrument or questionnaire or survey or tool or data collection AND
EMBASE	
Cochrane library	(3) EBP or evidence-based practice or EBM or evidence-based medicine or best evidence medical education or BEME or evidence-based nursing or research evidence or evidence-based health care or critical apprais* AND
Web of Science	
Informit - health database	(4) Teach* or learn* or educat* or curricul* or student* or train* or course* or workshop* or journal club* or program evaluat*
EQUATOR network	

* truncation symbol.

#### Information sources and search plan

Studies will be identified by searching electronic databases and reviewing the reference lists of included studies to identify further studies. The full list of included studies will be reviewed by the Expert Panel to identify any further studies.

#### Selection procedure

The initial search will be conducted independently by two researchers (AP and a person independent to this study) using the prospectively developed search protocol detailing the specific search terms with adequate translation for each database [20]. The results of the two independently completed searches will be compared and any disagreements will be resolved by discussion. If the protocol requires refinement, the process will be repeated.

#### Inclusion / exclusion criteria

 Studies will be eligible for inclusion in this review if they are:

• Published in peer reviewed journals irrespective of language of publication or publication date.

• Primary controlled trials defined as studies including a separate group for comparison (e.g. controlled trials; randomised controlled trials) which report original data for an educational intervention specific to developing knowledge and skills of EBP.

• Educational interventions within any teaching mode (face to face, online, group or individual) and must include at least one of the five steps of EBP.

• Any level of health professional training (undergraduate, postgraduate, continuing education courses).

• Secondary studies which meet the criteria defined in the PRISMA statement [12] to be classified as a systematic review or meta-analysis reporting primary studies of educational interventions specific to developing basic knowledge and skills of EBP (retained to assist identification of relevant primary studies).

Studies will be excluded if they:

• Describe or report evidence based guidelines or educational interventions specific to health conditions rather than educational interventions to develop skills and knowledge of EBP (for example, evidence based education for conservative management of hip osteoarthritis).

• Focus on educational interventions for facilitating learning of statistical concepts without at least one of the five key EBP steps.

• Report barriers, facilitators, attitudes, and behaviours relating to EBP without an educational intervention.

• Are narratives, letters and books providing recommendations or strategies for teaching skills in EBP.

• Describe or report the development of a survey instrument to gather information about EBP curricula/syllabi or courses without an EBP intervention to apply/test the instrument.

### Screening process

To ensure the selection process is consistent, the Doctoral Panel will collaboratively review the first 150 records to determine which articles meet the inclusion criteria based on title and abstract. Any issues and/or disagreements will be discussed and resolved by consensus. The Principal Investigator will then review titles and abstracts and remove any studies that do not clearly meet the inclusion criteria.

From the resultant list, further review will be conducted by two reviewers independently (AP and MTW) to determine the inclusion/exclusion of studies. The full text of relevant studies will be sought where the abstract is unavailable or where a decision about inclusion/exclusion cannot be made based on information provided in the abstract. The reference lists of included studies will be screened to identify additional publications. Disagreements between the reviewers will be resolved by a third independent reviewer. The complete list of controlled or higher level studies will be provided to the Expert Panel to consider whether any studies meeting the inclusion criteria have been omitted and these will be included as required.

#### Data extraction

All included studies will be accessed as full text, retrieved and stored electronically. A data extraction sheet will be developed prospectively based upon the data items used by Shaneyfelt, Baum, Bell, Feldstein, Houston, Kaatz, Whelan and Green. (2006), Flores-Mateo and Argimon (2007), Lewis, Williams and Olds (2011), McEvoy, Williams and Olds (2011) and the Cochrane Handbook “Checklist of items to consider in data collection” [21] (Table 2).

**Table 2:** **Proposed data extraction items (adapted from the Cochrane Handbook 2011)**[21]

	**Information to be extracted**
**Eligibility**	Reason for inclusion / exclusion
For studies retained in review	
**Descriptive**	Journal, author(s), citation and contact details, professional discipline of authors, year of publication, corresponding author and contact details, language of publication, country study undertaken, key words, study design.
**Participants**	Learners’ level of education, professional discipline, previous EBP exposure, sample size (including prospective sample size calculation), randomisation of sample, age, instructors’ qualification, experience in EBP.
**Intervention mode and delivery**	Type of intervention, duration, mode of delivery.
**Content**	Reference used for EBP, EBP steps described e.g. (ask, acquire, appraise, apply and assess).
**Evaluation**	Name and type of assessment method (e.g. exam, assignment, FRESNO), psychometric properties, whether a named test was used and/or modified, randomisation of intervention.
**Confounding issues**	Verbatim statements of issues confounding the EBP educational intervention or interpretation of the learning outcome.

Data are intended to be extracted in six domains;

1. Descriptive: (e.g. journal, year of publication, authors, professional discipline of authors, corresponding author, contact details, language of publication, key words, study design).

2. Participants: (e.g. “Learners” level of education, professional discipline, “teachers” qualification, experience in EBP, sample size).

3. Intervention mode and delivery: (e.g. face to face, on-line, group, individual, lecture, practical, journal club, discussion groups).

4. Content: (e.g. presence and detail of the five steps of EBP).

5. Evaluation: (e.g. methods and instruments).

6. Confounding issues: (e.g. verbatim statements of issues confounding the EBP educational intervention or interpretation of the learning outcome).

The data extraction sheet will be pilot tested for inter-rater reliability by two reviewers (AP and one member of the Doctoral Panel) with a random sample of 10% of included studies. For items where extraction or coding is inconsistent between reviewers (<80% agreement) [22], reviewers will convene to clarify discrepancies, the guidelines for data extraction will be revised and the data extraction sheet modified accordingly. The data items will then be double extracted for all included studies [22]. On completion of data extraction, data will be compared between extractors for consistency, analysed and reported descriptively (frequency of items reported in each domain).

#### Assessment of methodological quality and validity of included studies

The aim of this systematic review is to describe how EBP educational interventions have been reported rather than describing the efficacy of EBP educational interventions. As such assessment of methodological quality or external validity (generalisability of study and/or results) is not warranted [23]. It is possible that lower level designs (i.e. not RCT/controlled trials) may provide different descriptions of the educational intervention and report different items. To explore this possibility, data will be extracted from a random selection of excluded studies with lower level designs (pre-post without control and narrative reviews) and compared with the higher level studies (RCT/controlled trials) used during the reliability process (n = 15).

### Strategy for data synthesis

A narrative synthesis is planned. Descriptive synthesis of the findings from included studies will be structured around the six domains of Descriptive, Participants, Intervention mode and delivery, Content, Evaluation and Confounding issues. Summaries of the results from included studies will be provided and a list of all items reported by included studies will be compiled. The range, mean and standard deviation for reporting of items will be calculated for descriptive purposes.

### Outcomes

At the completion of the systematic review a list will be compiled containing all reported information items. This list will be reported using the six domains of (1) Descriptive, (2) Participants, (3) Intervention mode and delivery, (4) Content, (5) Evaluation and (6) Confounding issues and will include each specific item and the frequency each item was reported. This list will be used in stage 2 to ensure that all information items identified by the systematic review are provided to the Delphi participants to determine their importance and relevance for reporting.

The final outcome of the systematic review will comprise submission of a manuscript to disseminate the findings of the systematic review.

## Stage 2

### Delphi survey

#### Design and setting

**Ethics** Ethical approval has been obtained from the University of South Australia Human Research Ethics Committee (**protocol no. 25590)**

**Design** A Delphi survey will be conducted according to the checklist of 18 items described by Sinha et al. (2011) [24]. The Delphi process will comprise a series of four rounds of questionnaire, response and feedback until consensus is achieved [25]. The first round survey will commence with an open ended question and the three subsequent rounds will be undertaken providing feedback from the previous round and inviting further responses from participants [24]. It is intended that the Delphi will be informed by the systematic review undertaken in stage 1. Any items identified in the systematic review that are not included in the Delphi list at the completion of the second round will be added as ‘additional’ items for the third round of the Delphi. This will allow a further two rounds of review by participants for both items volunteered by the group as well as additional items derived from the systematic review, before the completion of the Delphi survey. Consensus will be defined ‘a priori’ as recommended by Sinha, Smyth and Williamson (2011) [24]. There are no current internationally accepted criterion standards to determine whether a consensus has been reached. For the purpose of this study consensus will be deemed to be achieved for each item of >80 per cent agreement indicating substantial to excellent agreement [26].

#### Participants

All corresponding authors of studies included in the systematic review and the editors of the journals in which these studies were published (stage 1) will be invited to participate in the Delphi survey (likely maximum number 110).

**Recruitment process** Invitation and participation in the Delphi survey will be completed via email which will outline the aim, likely time commitment and process of the Delphi survey [24]. Those who do not respond to the initial invitation will be emailed seven and 14 days after the initial invitation.

All participants will be allocated a random identification number for reporting and collation of the results. Demographic data regarding the participant’s profession, qualifications and contact details will be recorded. Participants will be invited to provide their name and consent to be acknowledged as a member of the Delphi panel in presentations / publications arising from this research. All participants who accept the invitation to participate in the Delphi survey will be invited to complete each and every Delphi round, regardless of participation in the previous round unless they indicate withdrawal from the Delphi.

**Procedure** The Delphi process will be conducted using an electronic survey format with embedded links to survey software (SurveyMonkey®). This software allows an unlimited number of participants, questions / responses and allows for export of responses to both Excel and SPSS formats.

#### First round

A brief preamble will be provided concerning the aim of the survey, definition of key terms, likely time commitment, plan for four rounds as well as the importance of completing all four rounds. As circumstances may have changed since initial recruitment, participants will be asked to contact the Principal Investigator (AP) if they wish to withdraw and will be removed from the list of participants.

The first round will commence with an example of a description of an educational process for facilitating knowledge and skills in EBP followed by an open ended question.

The proposed initial question is: 

“If you were reading a study which reported an educational process for facilitating foundation skills in evidence based practice (ask, acquire, appraise, apply and assess) what information about the INTERVENTION would you expect to be included?

Please list ALL the items of information that you would expect to be included by authors to describe any evidence based practice educational intervention. ”

There will be one reminder following each Delphi round which will be sent seven days after the dissemination of the survey. Participants will be provided a further seven days to respond and the Delphi round will close 14 days after the initial survey is sent.

All completed surveys will be analysed by the Principal Investigator. All responses will be downloaded verbatim to an Excel spread sheet and analysed using Excel and/or SPSS. Specific items within text responses to the open question will be identified and allocated by the Principal Investigator (and reviewed by the Research Team) into the six domains planned for data extraction within the systematic review: (1) Descriptive, (2) Participants, (3) Intervention mode and delivery, (4) Content, (5) Evaluation and (6) Confounding issues. All items that are not able to be clearly assigned to a domain will be discussed by the Doctoral Panel and a consensus decision for their allocation reviewed by the Expert Panel [24]. Once consensus has been reached concerning the domains and individual items within domains, frequency of responses will be calculated (per item for the total number of respondents).

#### First round analysis

A list of items within domains will be compiled. Frequencies will be calculated for all items and all participants.

#### Second round

Each item produced from the initial round will be allocated an identification number and randomised for frequency (within domains) [26]. To minimise the potential influence on the selection of items, the frequency with which specific items were provided by participants will not be reported to participants in the second round.

The randomly ordered list of items generated from the first round will be provided to all participants with instructions to rate the importance of each item on a Likert scale. A rating of zero meaning that the item is of limited importance and not required for reporting, up to a rating of 10 meaning that the item is of high importance and therefore essential for reporting.

The instruction for each participant will be:

“Here is a list of items which participants indicated should be reported when describing EBP educational interventions. For each item, please rate how important you think each item is for reporting an EBP educational intervention on the scale below.”

After completing the rating exercise, participants will have the opportunity to provide further items by responding to the following questions:

• If you rated any items <4 or >8, please provide a brief justification or reference to support your choice.

• Are there any other items that you believe should be reported when describing EBP educational interventions or teaching?

• Are there any comments you would like to add?

#### Second round analysis

The total number of completed surveys (number of participants) will be recorded and the rating for each item for each respondent downloaded to a spread sheet for descriptive analysis.

Likert scores will be designated into four categories. These categories, based upon the ranges used to define agreement for Cohen’s kappa, have been adapted for the Likert scale system on SurveyMonkey® (whole numbers only) [26].

The four categories according to Likert score (0–10) are:

• 0–4: Low importance. Item not included.

• 5–6: Moderate importance. Possible consideration for inclusion.

• 7–8: High importance. Likely to require inclusion.

• >8: Very high importance. Essential for inclusion.

Descriptive statistics [percent agreement, mean score, SD, range, mean absolute deviation from the median (MADM)] for each item and each category will be computed. To be considered to have met the consensus criterion, 80 per cent or more respondents will need to rate an item’s importance within the range for one category (low importance 0–4, moderate importance 5–6, high importance 7–8, very high importance > 8).

#### Third round

The list of items from the second round of the Delphi will be cross-checked against items derived from the systematic review (stage 1). If there are items from the systematic review which do not appear in the results of the second round survey, they will be added as ‘additional items’ for the participants to consider in round three. This entire list of items will be randomly ordered and will comprise the third round survey.

 Participants will be provided with descriptive feedback (percent agreement, mean, score, SD, range, MADM) for each item and each category along with any items which have achieved consensus. Any items that reach consensus will not be required for further comment and will be included in a separate page (screen) of the survey. All other items, including new items derived from participants from round two and additional items from the systematic review will be listed in random order. Participants will be asked to rate each item using the Likert scale.

After completing the rating exercise, participants will have the opportunity to provide further items by responding to the following questions:

• If you rated any items <4 or >8, please provide a brief justification or reference to support your choice.

• Are there any other items that you believe should be reported when describing EBP educational interventions or teaching?

• Are there any comments you would like to add?

#### Third round analysis

This will replicate the analysis from the second round including the number of participants from the second round and the rating scores for each item and any items which have achieved consensus.

#### Fourth round

This Delphi process has been prospectively planned to have four iterations. Feedback from the third round results will be included as part of the survey. The information provided in the feedback will include a list of all items generated from the preceding round and which items (if any) have achieved consensus. Any items that reach consensus will not be required for further comment and will be included on a separate page. All other items, including new items derived from round three, will be listed in random order and participants will be invited to rate each item using the Likert scale.

After completing the rating exercise, participants will be asked two final questions:

• Would you be interested in reviewing the draft of the reporting guideline and associated document?

• If you are currently undertaking an EBP educational strategy and plan to submit this for publication, would you be willing to pilot test the draft guideline?

#### Fourth round analysis

This will replicate the analysis from the previous two rounds. Items which do not reach the pre-determined level of consensus (>80% agreement) will be categorised based upon their mean rating score (0–4: Low importance. Item should not be included; 5–6: Moderate importance. Possible consideration for inclusion; 7–8: High importance. Likely to require inclusion and >8 Essential. Item included).

#### Outcome of fourth round

At the completion of the fourth round, all items derived from either the systematic review or the Delphi survey will have been described as either 1) item met consensus for inclusion in the reporting guideline or 2) item did not meet consensus but could be considered for inclusion in the reporting guideline, 3) item did not meet consensus and mean rating score suggests it should not be included in the reporting guideline.

This list of information items will form the basis for the content of the first draft of the GREET statement which will be compiled by the Research Team in stage 3.

## Stage 3 Development and pilot testing of the reporting guideline and explanation and elaboration document

The development of an explanation and elaboration paper to accompany the reporting guideline is of vital importance and yet is a common omission in the development and operationalization of most reporting guidelines [15]. The role of the explanation and elaboration document is to provide the background, rationale and justification for the guidelines as well as to provide examples for users regarding what information should be included and how to report this information. A detailed explanatory Explanation and Elaboration (E&E) paper is planned to be developed concurrently with the reporting guideline.

### Procedure

The Research Team will be convened via an email/website discussion to determine the proposed plan for the development of the GREET statement and E&E paper. The aim of this initial discussion will be to determine the best form of communication (email, internet chat site etc.), to nominate a chair person from the Expert Panel, to plan meetings, method of feedback (e.g. track changes), to provide a structure and work plan for writing the draft and method for resolving disagreements.

 Following this initial meeting the Principal Investigator in collaboration with the Doctoral Panel will draft the initial Guideline for Reporting EBP Educational interventions and Teaching (GREET) statement and E&E paper. The basis for each item will be described in the draft recommendations. This will include the origin of the item (systematic review or Delphi survey or both), the degree of consensus achieved in the Delphi survey and a brief rationale for inclusion. The draft document will be provided to the Expert Panel who will be convened in a Skype conference consensus meeting. The meeting will provide the opportunity for the Research Team to discuss the items to be included, and the layout and design of the GREET statement and E&E paper. The GREET statement and E&E paper will then undergo subsequent review and feedback via email or subsequent Skype conference call at the Expert Panels’ discretion until the final draft is considered complete. The draft GREET statement and E&E paper will then undergo review and pilot testing for the content, layout and wording of the document [15].

### Pilot testing of reporting guideline and explanation and elaboration document

#### Participants

Participants who have expressed interest at the completion of the fourth round of the Delphi survey in either reviewing the draft GREET statement and E&E paper or trialling the documents to report an EBP educational intervention will be contacted.

#### Procedure

The final draft of the GREET statement and E&E paper will be distributed to all interested participants. The participants will fall into two groups. One group will fulfil an editorial role by reviewing the guidelines to provide comments and suggestions regarding the content, structure, clarity and layout. The second group will include participants who have indicated that they are planning to report an EBP educational intervention and will be invited to trial the GREET statement and E&E paper to report their EBP intervention and to provide comments and feedback on the utility of these two documents. Participant feedback including clarification of any difficulties will be recorded verbatim and considered for incorporation into the checklist revisions [15].

#### Publication plan

• Publication 1: Study protocol.

• Publication 2: Systematic review.

• Publication 3: Delphi survey.

• Publication 4 & 5: GREET statement and E&E paper planning for simultaneous publication [15].

## Discussion

Over the past 25 years the philosophy and practice of EBP has evolved rapidly. This is reflected by the number of instruments developed to evaluate the efficacy of the teaching of EBP [8]. This rapid growth has resulted in heterogeneity of studies and inconsistencies in reporting of data. To address this problem, Simera, Moher, Hirst, Joey, Schulz and Altman (2010) [27] recommend that academic and other research institutions “promote and support accurate and transparent reporting of health research studies and the use of reporting guidelines…” Despite the recent rise in the number of reporting guidelines for health research, there are no reporting guidelines for educational interventions. The aim of this program of research is to develop a reporting guideline (GREET statement) and an explanation and elaboration (E&E) paper to enable the consistent and transparent reporting of EBP educational interventions and teaching.

Following a recent systematic review of reporting guidelines for health research, Moher, Weeks, Ocampo, Seely, Sampson, Altman, Schulz, Miller, Simera, Grimshaw and Hoey (2011) produced a guidance statement for developers of reporting guidelines. This guidance statement forms the backbone for this program of research. To develop a reporting guideline for health research, Moher, Schulz, Simera and Altman (2010) recommend undertaking ‘initial steps’, which include identifying the need for a guideline and reviewing the literature, followed by ‘pre-meeting activities’ which refer to the preparation required for the next stage, the ‘face to face consensus meeting’ where consensus is achieved. This ‘pre-meeting stage’ will often include a Delphi survey to generate a list of items to be further discussed at the ‘face to face consensus meeting’. This is then followed by the ‘post meeting activities’ which includes the development of the reporting guideline, E&E document and pilot testing and publication strategy. The ‘post-publication activities’ form the final stage in the development process and includes dealing with the feedback and criticism and updating of the guidelines.

The inclusion of the Expert Panel representing the fields of EBP education, research guideline development, epidemiology and journal editing will guide the development and testing processes used in this study. Engaging an expert panel is not a requisite step in the development process for reporting guidelines, however it provides a safeguard to ensure the entire development process is transparent and comprehensive [15].

 In this research protocol, the systematic review and Delphi consensus survey amalgamate the recommended ‘pre-meeting’ and ‘face-to face consensus meeting’ stages [15]. While a ‘face to face consensus meeting’ allows for a captive audience and the potential for clearer communication there are several possible limitations. The process can be time consuming and expensive, with previous reporting guidelines requiring 2–3 days for the initial face to face meeting and a recommended funding requirement of $75K for this meeting alone [15,28]. The travel required to attend a face to face meeting may preclude international participants thus limiting attendance and potentially introducing personal bias [24]. For these reasons, this protocol plans to use a Skype conference meeting in lieu of the recommended ‘face to face consensus meeting’ to discuss the results of the Delphi consensus survey. The Delphi survey is intended to be used as the primary means of achieving consensus with the Skype conference to discuss and finalise the content and proposed layout of the GREET statement and E&E paper.

With respect to the Delphi consensus stage, this research protocol is based upon the recommendations from a recent systematic review as the consensus method [24]. There are currently no internationally accepted criterion standards to determine the number and composition of participants for a Delphi survey [24]. Previous Delphi surveys have used as few as 10 to as many as 1685 participants [29]. Delphi surveys have previously been used in the development of nine reporting guidelines for health research [22,30] with the number of participants (reported by only seven studies) ranging from 11 (CONSORT) [11]to more than 50 (SQUIRE) [31]. The potential number of participants planned for this program of research (likely maximum of 110 participants) is larger than has been used in previous guideline development because all corresponding authors of articles included in the systematic review (encompassing researchers and educators in the field of EBP education, statisticians and epidemiologists) will be invited to participate in the Delphi survey [15].

 Traditionally the Delphi survey uses a minimum of three and a maximum of four rounds or until consensus is achieved [25]. The number of rounds used in Delphi surveys to date varies from as few as two [12,28,30] to as many as six [32]. Four rounds will be used in the proposed Delphi survey to maximise open ended survey responses in the first two rounds, and allow for additional items identified in the systematic review to be introduced in round three. Commencing the Delphi survey with an open ended question rather than using a pre-determined list of items will ensure that the researchers’ views are not imposed upon the participants, thus enabling the participants to suggest all possible outcomes for consideration [24].

Previous studies have used a systematic review to generate a pre-determined list of items to open a Delphi survey [24]. In the current program of research, a systematic review will be used to inform the Delphi survey with a list of items identified in the review incorporated into the Delphi at the completion of the second round. This should provide sufficient transparency and time over two further Delphi rounds to determine the opinion of the panel regarding the importance of the items and whether or not they should be included as reporting requirements.

 It is recommended that the level of consensus for the Delphi survey is both clearly defined and declared ‘a priori’ [24]. However there are no current internationally accepted criterion standards to determine whether a consensus has been reached [24]. Previous Delphi studies used in the development of reporting guidelines for health research do not report adequate detail to provide a guide for this study. Only two of the Delphi surveys used in the development of reporting guidelines for health research reported the level of agreement required to achieve consensus [30,33] and consensus was not declared ‘a priori’ for any of the Delphi surveys [22,30]. Consensus can be defined as a level of agreement and calculated using a mathematical formula or equation (e.g. percentage agreement, kappa analysis). However a consensus opinion is not necessarily the ‘correct’ opinion nor is it necessarily an accurate answer to a question [34]. For the purpose of this study consensus will be defined ‘a priori’ and deemed to be achieved for each item using descriptive statistics of >80 per cent agreement [26].

 The importance of reporting guidelines are well recognised and the benefits of reporting guidelines that have been developed using robust and widely accepted methodologies include improving the standard and transparency of reporting, assisting peer reviewers and editors to strengthen manuscripts and potentially streamlining research funding applications [14,22]. The advantages of E&E documents, previously described as an ‘essential element’ in the development of reporting guidelines, are less widely recognised, and as a result, these documents have been largely overlooked by guideline developers. The E&E document provides the background and justification for the reporting guideline and can assist users by providing examples of how to report information [15]. There are very few E&E documents available in comparison to the large number of reporting guidelines available, with fewer than 15 per cent of reporting guidelines providing an accompanying E&E document [15]. The development of an E&E paper forms an essential part of the final stage of this program of research.

To determine how useable documents are, requires testing in the real world and Moher, Schulz, Simera and Altman (2010) recommend considering a pilot testing process in the development of the reporting guideline. However most reporting guidelines do not include a pilot testing process, with the minority of reporting guidelines (n = 11, 13.6%) reporting a pilot testing process [22]. Pilot testing is planned for this program of research to determine whether the GREET statement and E&E paper are written at an appropriate level and whether they provide sufficient instruction for potential users. This will be achieved by gaining two perspectives from participants; an editorial perspective and a user perspective. The Delphi participants will be invited to review the documents and provide feedback with respect to the layout, wording and structure of the documents. Researchers will be invited to use the GREET statement and E&E paper to report their educational interventions and to provide feedback on the usability of the GREET statement and E&E paper.

 There are knowledge frameworks other than EBP, but this decision making paradigm is rapidly becoming part of accreditation requirements for entry-level and post graduate health programs in educational institutions and various health care registration bodies. There is a rapidly growing body of research concerning educational practices used to teach and facilitate knowledge and skills in EBP, hence the consensus papers concerning what should be taught in EBP (Sicily 1) and the development of specific outcome instruments for use in assessing EBP foundation knowledge, skills, behaviours and attitudes (Sicily 2). There are philosophical differences in terms of which frameworks are appropriate for exploring educational interventions and definitions of what evidence should be considered. Regardless of which framework is proposed or accepted, there is value in having a guideline for use when reporting the educational intervention**.** This guideline would allow consistency in describing the educational intervention irrespective of the study design / methodological approach used by researchers.

 The development of the GREET statement and E&E paper will be the culmination of a development process that is both robust and applies widely accepted methodologies. This will be the first reporting guideline for EBP educational interventions and as such is an important milestone. As the reporting of EBP educational interventions is inconsistent at best, improving the standard of how educational research is conducted, reported and published is imperative. Developing the GREET statement will provide a reporting guideline for EBP educational interventions that can be used in conjunction with existing guidelines for research design to potentially benefit all stakeholders in EBP education: researchers, educators, editors, reviewers and students.

## Competing interests

Dr Moher is supported by a University Research Chair. Dr Moher is a member of the EQUATOR executive committee.

## Authors’ contributions

AP, LKL, MPM, MTW, conceived the idea and developed the draft protocol. PG, DM, JG, JT, MH, revised, developed and reviewed the final protocol. All authors read and approved the final manuscript.

## Pre-publication history

The pre-publication history for this paper can be accessed here:

http://www.biomedcentral.com/1472-6920/13/9/prepub
